# Cyclic Stretch Facilitates Myogenesis in C2C12 Myoblasts and Rescues Thiazolidinedione-Inhibited Myotube Formation

**DOI:** 10.3389/fbioe.2016.00027

**Published:** 2016-03-21

**Authors:** Ya-Ju Chang, Yun-Ju Chen, Chia-Wei Huang, Shih-Chen Fan, Bu-Miin Huang, Wen-Tsan Chang, Yau-Sheng Tsai, Fong-Chin Su, Chia-Ching Wu

**Affiliations:** ^1^Department of Cell Biology and Anatomy, National Cheng Kung University, Tainan, Taiwan; ^2^Institute of Basic Medical Sciences, National Cheng Kung University, Tainan, Taiwan; ^3^Department of Microbiology, College of Medicine, National Taiwan University, Taipei, Taiwan; ^4^Department of Occupational Therapy, National Cheng Kung University, Tainan, Taiwan; ^5^Department of Occupational Therapy, I-Shou University, Kaohsiung, Taiwan; ^6^Department of Biochemistry and Molecular Biology, National Cheng Kung University, Tainan, Taiwan; ^7^Institute of Clinical Medicine, National Cheng Kung University, Tainan, Taiwan; ^8^Department of Biomedical Engineering, National Cheng Kung University, Tainan, Taiwan; ^9^Medical Device Innovation Center, National Cheng Kung University, Tainan, Taiwan

**Keywords:** myogenesis, PPAR, stretch, C2C12, myotube formation

## Abstract

Thiazolidinedione (TZD), a specific peroxisome proliferator-activated receptor γ (PPARγ) agonist, was developed to control blood glucose in diabetes patients. However, several side effects were reported that increased the risk of heart failure. We used C2C12 myoblasts to investigate the role of PPARs and their transcriptional activity during myotube formation. The role of mechanical stretch during myogenesis was also explored by applying cyclic stretch to the differentiating C2C12 myoblasts with 10% strain deformation at 1 Hz. The myogenesis medium (MM), composed of Dulbecco’s modified Eagle’s medium with 2% horse serum, facilitated myotube formation with increased myosin heavy chain and α-smooth muscle actin (α-SMA) protein expression. The PPARγ protein and PPAR response element (PPRE) promoter activity decreased during MM induction. Cyclic stretch further facilitated the myogenesis in MM with increased α-SMA and decreased PPARγ protein expression and inhibited PPRE promoter activity. Adding a PPARγ agonist (TZD) to the MM stopped the myogenesis and restored the PPRE promoter activity, whereas a PPARγ antagonist (GW9662) significantly increased the myotube number and length. During the myogenesis induction, application of cyclic stretch rescued the inhibitory effects of TZD. These results provide novel perspectives for mechanical stretch to interplay and rescue the dysfunction of myogenesis with the involvement of PPARγ and its target drugs.

## Introduction

Muscle tissue is packed with an ordered array of myofibers composed of tightly bundled, long, cylindrical multinucleated muscle cells. The repeated actin–myosin myofibrils are the contractile units of the muscle filaments that slide during contraction. The effect of mechanical stimulation on cell homeostasis and development, which are critical factors in tissue maintenance, repair, and regeneration, has drawn a lot of attention (Su et al., 2011). In myogenesis, the constant mechanical and chemical stimulations drive the myogenic cells to become stable postmitotic multinucleated muscle (Pownall et al., 2002). Mechanical stretch can alter the organization of stress fibers in myocytes (Yamazaki et al., 1996; Hornberger et al., 2005; Saygili et al., 2007), smooth muscle cells (Simpson et al., 1994), and endothelial cells (Kaunas et al., 2005). Skeletal muscle has the ability to restore its cellular architecture after injury; however, myogenesis involves a complex sequence of molecular events that is particularly relevant to human diseases, such as inherited myopathies, systemic metabolic diseases, and common cardiovascular disorders (Bassel-Duby and Olson, 2006).

Type 2 diabetes mellitus (DM), accounting for approximately 90% of the diabetic population, is a metabolic disorder caused by insulin resistance. Thiazolidinedione (TZD) and rosiglitazone (RSG) are insulin sensitizers and are used as second-line drugs if the first-line drug – metformin – fails to control DM. Peroxisome proliferator-activated receptors (PPARs) are a group of ligand-dependent nuclear receptors with three isoforms: PPARα, PPARδ, and PPARγ. The different PPARs form a complex with cofactors to regulate their downstream target genes with diverse functions in different tissues (Rosen and MacDougald, 2006). TZD drugs work as PPARγ agonists to bind its receptors in fat cells and render the cells more responsive to insulin in DM patients. Although TZD-related drugs showed promising results in maintaining blood sugar levels, an increased incidence of heart disease and death has been reported in patients prescribed RSG (Steven and Nissen, 2007; Chen et al., 2012). A safety alert for RSG had been released by the U.S. Food and Drug Administration regarding its side effects with respect to cardiovascular risk (Graham et al., 2010; Komajda et al., 2010). Cautions for prescribing and administering TZD or RSG have been suggested, although the pros and cons of using these drugs are not yet conclusive (Kaul et al., 2010).

Exercise and dietary habits help to manage the initial stage of DM (Raina Elley and Kenealy, 2008). To study mechanical responses in cultured cells, the cells are maintained on the extracellular matrix-coated elastic membrane, which is then stretched to observe the cellular responses (Su et al., 2011). Application of cyclic stretching (1 Hz) to mouse preadipocyte 3T3-L1 cells inhibited adipocyte differentiation through extracellular signal-regulated kinase (ERK)-mediated downregulation of PPARγ2 (Tanabe et al., 2004). Cyclic stretching (10% at 0.5 Hz) also induced transforming growth factor β1-Smad signaling to inhibit adipogenic markers in human umbilical cord perivascular cells (Turner et al., 2008). C2C12 cells, a mouse-derived myoblast cell line (Yaffe and Saxel, 1977; Blau et al., 1985), is an ideal *in vitro* model for studying the molecular basis of myogenesis. Mechanical stretch activates rapamycin and adenosine monophosphate-activated protein kinase signaling (Nakai et al., 2015), phosphorylates Src-mediated myogenesis (Niu et al., 2013), and inhibits Toll-like receptor 3 autoantigens associated with inflammatory myopathy in C2C12 myoblasts (Chen et al., 2013). The *in vitro* ectopic expression of PPARγ in C2C12 cells induced their transdifferentiation into adipocytes (Hu et al., 1995). However, the influence of PPARs and their transcriptional activity when subjecting C2C12 myoblasts to mechanical stretch remains unknown. Furthermore, understanding the effect of RSG treatment on myogenesis dysfunction and intracellular PPARγ signaling during stretch might provide a comprehensive overview of drug usage to facilitate prevention of side effects in early DM. The current study investigated the role of PPARs and their transcriptional activity during different responses of myogenesis to stretch and/or RSG treatments. We discovered that cyclic stretch promoted myogenesis by decreasing the PPARγ protein expression and promoter activity that could prohibit the inhibitory effects of PPARγ agonist drugs.

## Materials and Methods

### Cell Culture and Differentiation

Mouse C2C12 myoblasts were purchased from the American Type Culture Collection (ATCC, VA, USA) and routinely cultured in Dulbecco’s modified Eagle’s medium (DMEM) (Invitrogen, Carlsbad, CA, USA) with 10% fetal bovine serum (Hyclone, Logan, UT, USA) at 37°C and 5% CO_2_. The undifferentiated myoblasts were maintained in subconfluent conditions to avoid differentiation. To evaluate the response of myogenesis and myotube formation to biochemical factors, the C2C12 cells (3 × 10^5^ cells) were seeded on 6-cm culture dishes coated with type I collagen (BD Biosciences, Franklin Lakes, NJ, USA), rinsed twice with phosphate-buffered saline (PBS) after cells reached monolayer confluency, and then replenished with fresh growth medium (GM), serum-free (SF) DMEM, or myogenesis medium (MM, DMEM containing 2% horse serum). Myotube formation was evaluated on phase-contrast microscopy (Olympus) images acquired at different time points. The myotube number per square centimeter and the average length of myotubes were quantified using ImageJ software to indicate the efficiency of myogenesis. Length of myotube were carefully measured by drawing line along the long axis of myotube between two myotube tips and convert the pixels into the unit of length in ImageJ software.

### Mechanical Stretch

A stretch system (STREX Cell Stretching system, ST-190; B-Bridge International, Inc., Cupertino, CA, USA) was used to apply cyclic uniaxial stretch with the deformation of 10% and frequency at 1 Hz (10%, 1 Hz). The C2C12 cells (2 × 10^5^ cells) were seeded in silicon stretch chambers coated with type I collagen. The C2C12 cells reached 90–100% confluency after being cultured in regular GM for 24 h. The cells were then rinsed with PBS, replenished with GM or MM, and incubated for an additional 24 h to initiate myogenesis induced by biochemical factors. The role of mechanical factors in myogenesis was tested by subjecting these cells to cyclic stretch for 1 h and then collecting the cell lysates.

### PPAR Drug Treatments and Luciferase Reporter Assays

The TZD, RSG, CAY10599, GW9662, WY14643, MK886, and MG132 reagents were purchased from Cayman Chemical (Ann Arbor, MI, USA). All reagents were dissolved in ethanol, except GW9662, which was dissolved in dimethyl sulfoxide for the stock solution and then diluted 1:10 in ethanol to achieve a concentration of 1 μM. To measure the PPAR promoter activity, we used a PPAR response element (PPRE) reporter conjugated to luciferase kindly provided by Drs. K. W. Kinzler and B. Vogelstein (Johns Hopkins University, Baltimore, MD, USA) (Liou et al., 2008). The C2C12 cells were transfected with the PPRE constructs using Lipofectamine 2000 (Invitrogen) for 24 h, washed with PBS, and then replenished with the medium containing the relevant drug. For measuring PPRE transcriptional activity during cyclic stretch, transfected C2C12 cells were transferred to the stretch chamber for 24 h and incubated for an additional 24 h with a different medium. PPRE activity was measured by collecting cells after they were subjected to cyclic stretch for 1 h. Luciferase activity was measured in cell lysates using the firefly luciferase kit (Promega, Madison, WI, USA) and a Sirius luminometer (Berthold Detection System; Berthold Technologies, Bad Wildbad, Germany) to measure the emitted light. The transcriptional activity of PPRE was quantified as relative light units per microgram of protein and expressed as fold change normalized to a control group.

### Western Blotting

The C2C12 myoblasts were treated with different biochemical and/or mechanical stimuli for specific times. Protein expression was assessed by collecting the cell lysates after rinsing cells twice with cold PBS and lysing with RIPA buffer containing protease inhibitors (Liu and Brooks, 2012). Briefly, cell lysate supernatants containing 30 μg total protein were transferred to nitrocellulose membranes (Bio-Rad, Hercules, CA, USA) following SDS-PAGE on 10% cross-linked gels. The membranes were blocked by 5% dry milk in Tris-buffered saline with 0.1% Tween 20 for 90 min. Antibodies for phospho-ERK1/2 (1:2000 dilution; Cell Signaling Technology, Danvers, MA, USA), ERK (1:1000; Santa Cruz Biotechnology, Santa Cruz, CA, USA), phospho-c-Jun N-terminal kinase (JNK) (1:1000; Cell Signaling Technology), JNK (1:1000; Santa Cruz Biotechnology), phospho-Akt (1:1000; Cell Signaling Technology), Akt (1:1000; Santa Cruz Biotechnology), myosin heavy chain (MHC) (1:4000; Sigma-Aldrich, St. Louis, MO, USA), α-smooth muscle actin (α-SMA) (1:4000; Sigma-Aldrich), PPARα (1:1000; Thermo Fisher Scientific, Waltham, MA, USA), PPARδ (1:1000; Cayman Chemical), PPARγ (1:500; Cell Signaling Technology), and β-actin (1:10000; Sigma) were incubated at 4°C overnight for evaluation of protein expression levels. The bound primary antibodies were detected using appropriate secondary antibodies coupled to horseradish peroxidase (Santa Cruz Biotechnology) and an enhanced chemiluminescence detection system (Pierce Biotechnology, Rockford, IL, USA).

#### Immunofluorescence Staining

To investigate the protein expression of myogenic markers, we used immunofluorescence staining to determine the myogenesis in C2C12 during different medium treatments. Briefly, cells were rinsed with PBS twice and fixed by 4% paraformaldehyde (Sigma-Aldrich) after various treatments. Then, the 0.1% Triton X-100 was used for cell membrane permeabilization prior the overnight hybridization of primary antibodies. The MHC and α-SMA were the same antibodies used in Western Blotting for indicating myogenesis. The FITC-conjugated secondary antibodies were used to visualize the myotube structure. The rhodamine phalloidin was used to indicate the cytoskeleton structure for stress fiber in all undifferentiated and differentiated C2C12 cells. Cell nucleus was identified with 4, 6-diamidino-2-phenylindole (DAPI) staining.

### Statistical Analysis

All experiments were repeated at least three times, and the data are expressed as means ± SDs. Statistical analysis was performed using one-way analysis of variance and the Scheffé *post hoc* test. Values of *p* < 0.05 were considered statistically significant.

## Results

### Myogenesis Decreased PPARγ Protein and Transcriptional Activity

**Figure 1:**
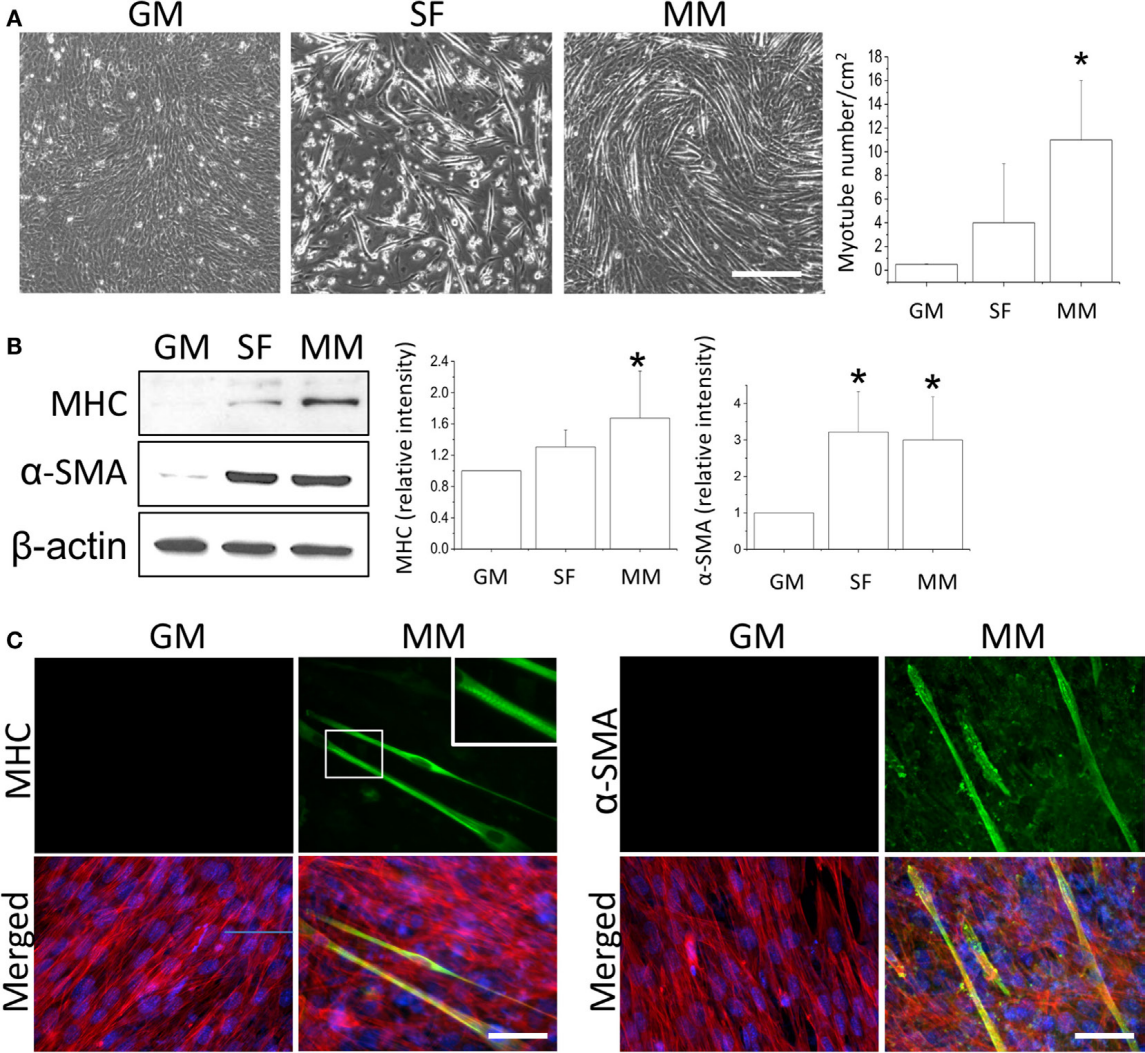
**Monolayers of C2C12 myoblasts were switched to growth medium (GM), serum-free medium (SF), or myogenic medium (MM) for 5 days, and myotube formation was then quantified (A)**. Induction of myogenesis was confirmed by the increase in myogenic protein markers myosin heavy chain (MHC) and α-smooth muscle actin (α-SMA) **(B)**. The confluency of C2C12 cells was demonstrated by immunofluorescence staining of cytoskeleton (red color for stress fiber) and nucleus (blue color for DAPI) in both GM and MM **(C)**. The mature myotube structure was visualized using MHC (green color, left panel) and the expression of early myogenesis marker was indicated by α-SMA (green color, right panel) after MM induction for 5 days. The striation of mature myotube was observed by MHC staining (zoom-in image). *Significant difference compared with GM (*p* < 0.05). Scale bar for **(A)**: 200 μm. Scale bar for **(C)**: 50 μm.

**Figure 2:**
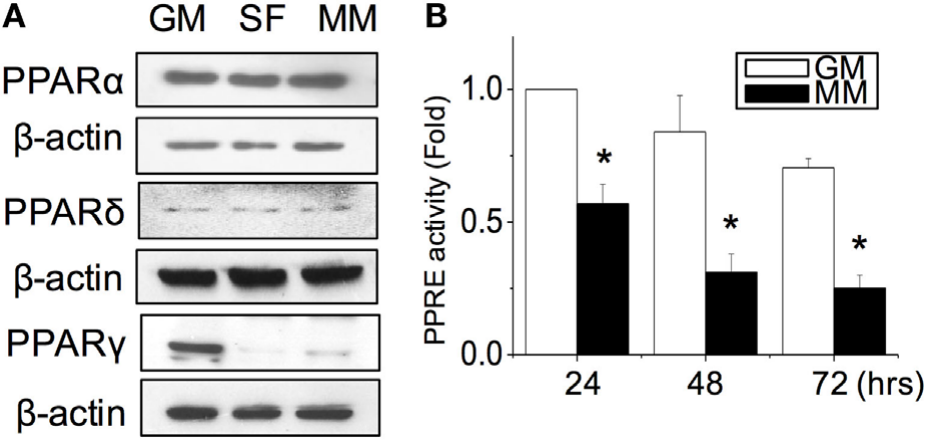
**PPARγ protein expression was decreased during myogenesis (A)**. The transcriptional activity of PPARs was decreased as measured by the promoter luciferase reporter construct of the PPAR-responsive element (PPRE) **(B)**. *Significant difference compared with GM (*p* < 0.05).

After the C2C12 myoblasts reached monolayer confluency, the switch to SF for 72 h not only induced some myotube formation but also caused cell death (rounding up and floating) (Figure 1A, SF). The MM significantly induced cell fusion into myotubes and increased the number of myotubes (Figure 1A). Successful myogenesis was confirmed by an increase in MHC and α-SMA protein expression after culturing C2C12 in MM for 72 h (Figure 1B). Immunofluorescence staining further demonstrated the myotube structures in myogenesis-specific markers, especially for the strip pattern of MHC staining, after MM induction for 72 h (Figure 1C, green color). The multi-nucleus structure after cell fusion to form the myotube can also be visualized by DAPI staining (Figure 1C, blue color). The intracellular stress fiber (Figure 1C, red color) was stained to indicate the cytoskeleton of all C2C12 cells. To investigate the role of PPARs in myogenesis, their protein expression was assessed, and a decrease in PPARγ was observed in C2C12 cells treated with SF or MM (Figure 2A). The protein expression of PPARα and PPARδ did not change after replacement with different culture medium. The PPRE promoter activity showed continuous decreases after switching to MM for 24, 48, and 72 h (Figure 2B). These results suggested the involvement of PPARγ and its transcriptional activity during biochemical-induced myogenesis. The confluent C2C12 cells cultured in MM had decreased PPAR transcriptional activity leading to a reduction of PPARγ protein.

### Cyclic Stretch Enhanced Myogenesis and Facilitated Suppression of PPARγ Expression

**Figure 3:**
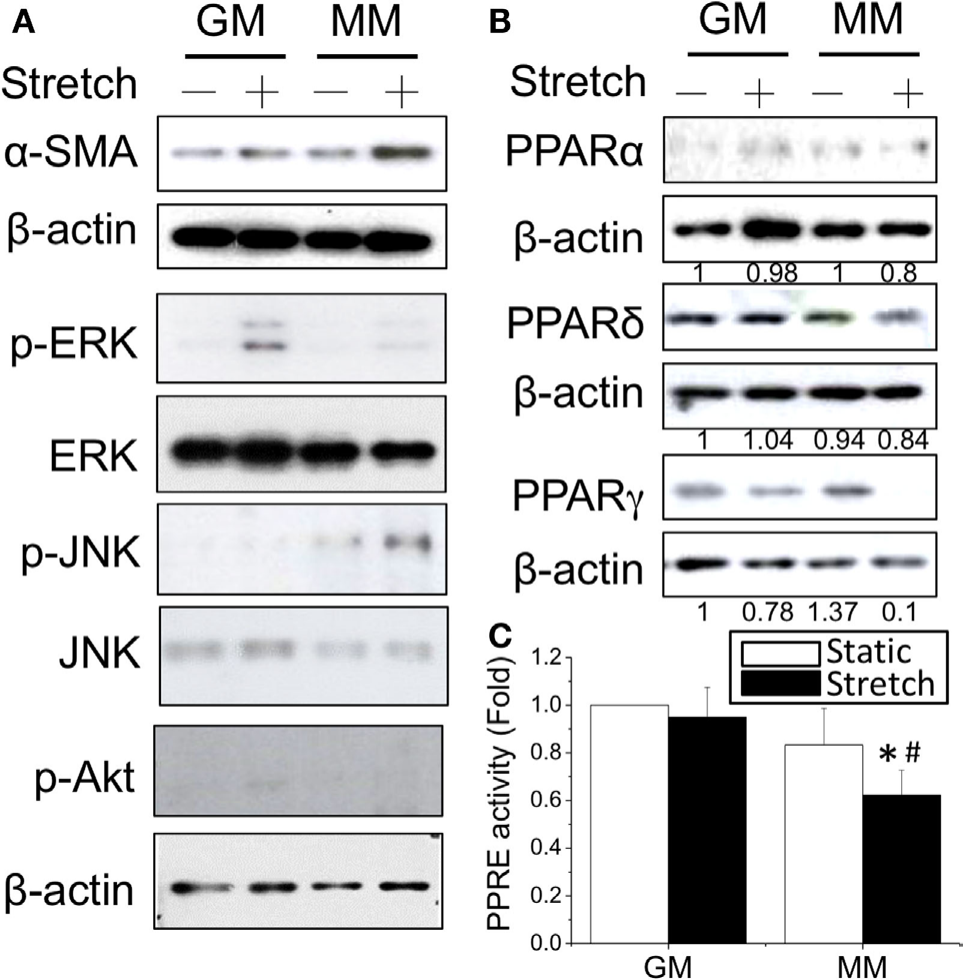
**Application of cyclic stretch (10% strain at 1 Hz for 1 h) in MM with further induced myogenesis as indicated by increased α-SMA expression (A)**. The phosphorylation of JNK was induced by applying stretch in MM, whereas ERK phosphorylation was observed when stretch was applied in GM. A decrease in PPARγ protein expression was also found during stretch-induced myogenesis in MM **(B)**. The number below each lane indicates the quantified fold change with normalized to GM static condition and its individual β-actin. A further decrease of PPRE promoter activity was detected when applying cyclic stretch **(C)**. *Significant difference compared with GM (*p* < 0.05). ^#^Significant difference compared with MM under static conditions (*p* < 0.05).

Since the mechanical stimulation induced myogenic differentiation, we were interested to examine how cyclic stretch interacted with biochemical factors to influence the intracellular signals and PPAR expression during myogenesis. The application of cyclic stretch for 1 h facilitated myogenesis as indicated by increasing α-SMA expression in MM but not in GM (Figure 3A). The stretch induced different mitogen-activated protein kinase (MAPK) signals in GM and MM. In regular GM, the cyclic stretch increased the phosphorylation of ERK (p-ERK). The C2C12 cells in MM also showed minor ERK phosphorylation, but the major MAPK signal triggered by stretch was JNK phosphorylation. The expression level of PPARγ protein was slightly decreased in GM, but almost abolished when applying stretch in MM (Figure 3B). The cyclic stretch also decreased the PPRE promoter activity in MM (Figure 3C). These data indicate the common inhibition of PPARγ during myogenesis by both biochemical and mechanical stimulation. Under biomechanical induction, the mechanical stretch further promotes myogenesis.

### PPARγ Agonist Inhibited Myogenesis and Myotube Formation

**Figure 4:**
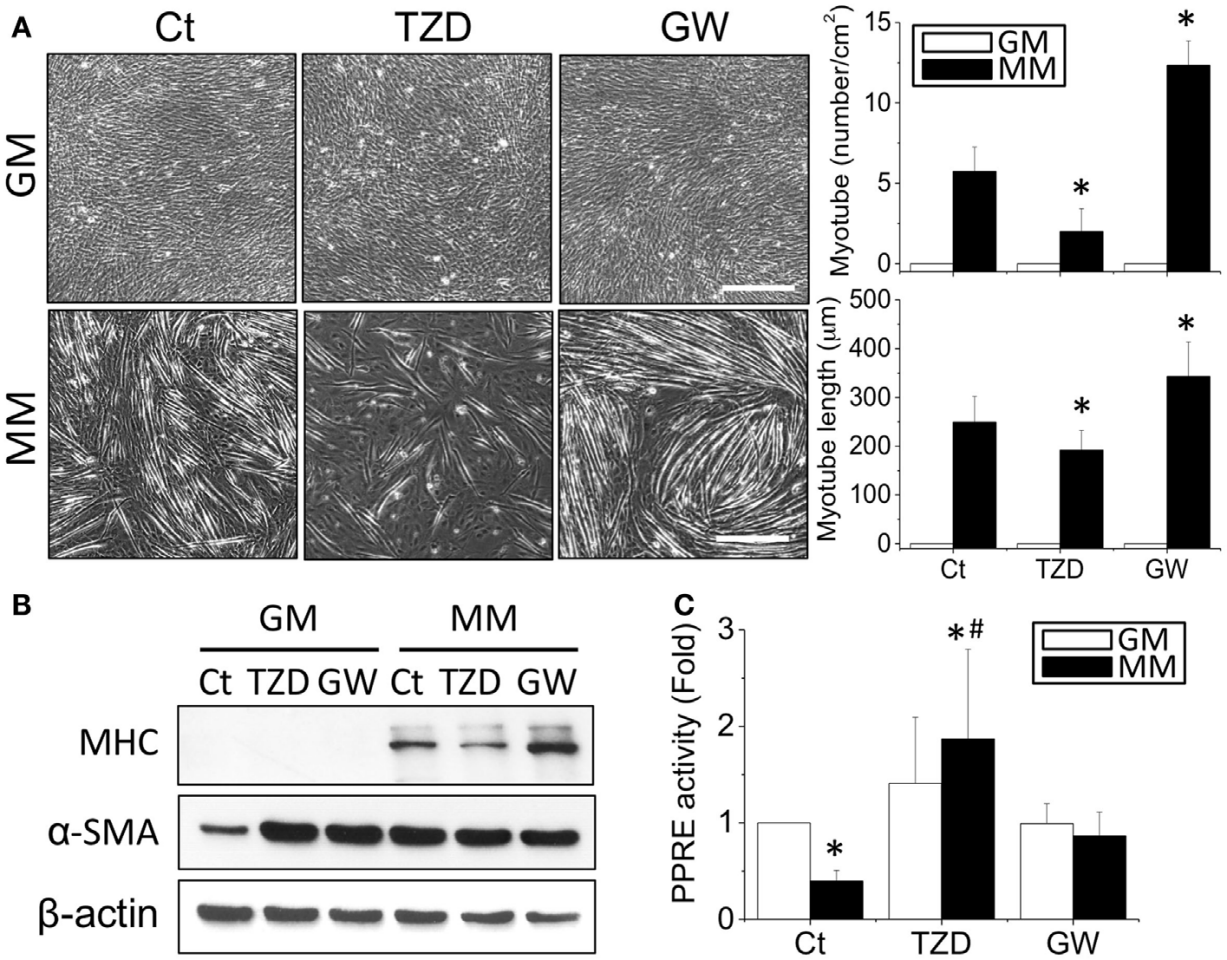
**The addition of TZD (PPARγ agonist) abolished the MM-induced myotube formation, but GW9662 (GW, PPARγ antagonist) increased the number and length of myotubes (A)**. The effect of PPARγ on myogenesis was confirmed by MHC protein expression, which was decreased with TZD and increased with GW treatment **(B)**. The administration of TZD significantly increased PPRE promoter activity **(C)**. *Significant difference compared with GM without drugs (Ct) (*p* < 0.05). ^#^Significant difference compared with MM without drugs (Ct) (*p* < 0.05).

The effects of TZD on myogenesis were then assessed by treating C2C12 cells with 10 μM TZD in GM or MM. Since TZD is a PPARγ agonist, the opposite effect of decreasing PPARγ was also tested by adding GW9662 (1 μM), a PPARγ antagonist. After 72-h culture, the phase images were taken randomly at five areas in each treatment and measured the number and length of myotube using ImageJ software. In MM, TZD significantly inhibited myotube formation, whereas GW9662 increased the number and length of myotubes (Figure 4A). The inhibition of myogenesis by a PPARγ agonist was also observed with administration of RSG, another member of the TZD class of drugs (Figure S1 in Supplementary Material). The effects of PPARγ agonists and antagonists in myogenesis were confirmed by decreasing and increasing MHC protein expression, respectively (Figure 4B). However, altering PPARγ signaling in biochemical-induced myogenesis using TZD or GW9662 did not change the α-SMA expression levels. The PPRE promoter assay showed that the reduction of transcriptional activity in MM was reversed by adding TZD to the MM (Figure 4C). To assess whether other PPARs also play a role in the myogenic process, C2C12 cells were treated with a PPARα agonist (10 μM WY14643 or ciprofibrate) or antagonist (10 μM MK886) for monitoring the degree of myogenesis (Figure S2 in Supplementary Material). There were no significant differences in MHC expression after administration of the drugs related to PPARα signaling.

### Stretch Prevented the Inhibitory Effect of PPARγ Agonist on Myogenesis

**Figure 5:**
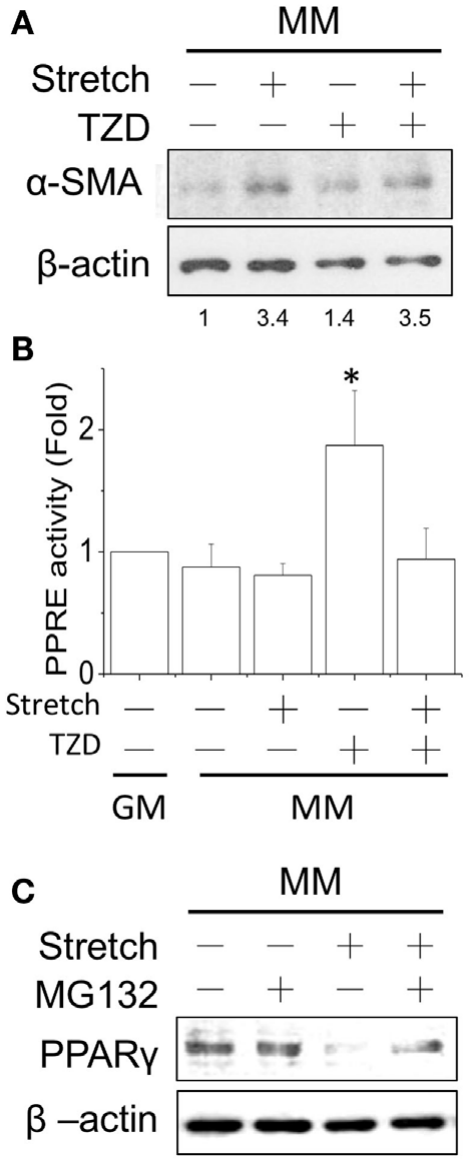
**After applying the cyclic stretch for 1 h, α-SMA protein expression was increased with TZD treatment in MM (A)**. The number below each lane indicates the quantified fold change with normalized to static condition without TZD and its individual β-actin. The mechanical stretch also inhibited the TZD-induced PPRE promoter activity **(B)**. The decreased PPARγ protein expression can be reversed by adding the protease inhibitor MG-132 during the cyclic stretch **(C)**. *Significant difference compared with GM without drugs (*p* < 0.05).

Since cyclic stretch promoted myogenesis in MM, we further tested the interactions of mechanical stretch and TZD treatments. The TZD was added to the MM after C2C12 cells were transferred to the stretch chamber for 24 h and then incubated for an additional 24 h. Compared with the stretch induction in MM, TZD treatment lowered α-SMA expression, and the application of stretch for 1 h under TZD treatment slightly prevented this inhibitory effect (Figure 5A). The prevention of TZD-reduced myogenesis was demonstrated by blocking PPRE promoter activity after the cyclic stretch (Figure 5B). Since mechanical stretch promoted myogenesis via a decrease in PPARγ, the proteasome inhibitor MG-132 was added to prevent ubiquitin-dependent protein degradation. Our results showed that MG-132 restored the decrease of PPARγ after stretch (Figure 5C). Consistent with our hypothesis that PPARγ activation would inhibit myogenesis, when cells were treated with TZD to evoke PPARγ expression, myogenesis was inhibited, and the application of mechanical stretch rescued this side effect of TZD.

## Discussion

In the current study, we used a simple myotube formation platform of C2C12 myoblasts to discover the role of PPARγ and its agonist in inhibiting myogenesis, which may explain in part the side effects of TZD administration. We found that the intracellular PPARγ levels were crucial in the progression of myogenesis. Lower PPARγ levels promoted myogenesis (Figure 1), whereas elevation of PPARγ levels inhibited cell differentiation (Figure 4). PPARα is found mainly expressed in tissues with a high metabolic rate, such as liver, muscle, kidney, and heart (Issemann and Green, 1990; Braissant et al., 1996). The expression of PPARγ is abundant in the intestine and adipose tissue (Tontonoz et al., 1994; Mansén et al., 1996), whereas PPARδ is expressed ubiquitously (Braissant et al., 1996). When DM patients use the TZD class of drugs to sensitize insulin, the increase in PPARγ protein expression in myogenic cells reduces its benefits. The inhibition of myogenesis causes major problems in muscle homeostasis, which may manifest not only in skeletal but also in cardiac muscles. In striated muscles, both the skeletal and cardiac myocytes repeatedly encounter stretch stimulation. Their strategies to cope with these mechanical signals may be conserved in evolution. The administration of a PPARγ activator, such as TZD or RSG, may block the regenerative potential in muscle progenitor cells. For elderly patients who may be more prone to muscle injuries, the therapeutic strategies of using TZD drugs to treat DM may be harmful with respect to muscle regeneration. However, cardiac hypertrophy caused by mechanical overload can be inhibited by increasing the PPARγ levels with treatments of TZD drugs or 15-deoxy-Δ^12,14^-prostaglandin J_2_ (Yamamoto et al., 2001; Yue Tl et al., 2001). Taken together with those of others in the literature, our findings allow us to highlight the importance of PPARγ in myogenesis. However, manipulation of PPARγ levels should be undertaken more carefully while considering the complexity of diseases when using TZD drugs.

Cells in skeletal muscle are constantly receiving mechanical and biochemical signals for developing highly differentiated muscle fibers. Stretch can enhance myogenesis and increase muscle mass (Goldspink et al., 1992) and, thus, is important in rehabilitation after muscle injury. In the hypothesis of mechanotransduction, the intracellular signal transduction initiates the mechanical signals that are transmitted from the extracellular matrix through membrane receptors, such as integrin or anchorage points linking to the cytoskeleton (Chien, 2007). Not only the decreases in PPARγ and its transcriptional activity under cyclic stretch but also other intracellular signaling pathways are involved in stretch-induced myogenesis. In the current study, we found that JNK was phosphorylated when facilitating myogenesis using a combination of biochemical and mechanical stimuli (Figure 3A). Uniaxial cyclical stretch can induce myoblast proliferation via a cyclooxygenase-2-dependent mechanism (Otis et al., 2005). The upregulation of nuclear factor-κB transcriptional activity was also found during stretch-induced myogenesis through the phosphorylation of p38 and RelA signaling (Ji et al., 2010). The addition of PPARγ ligands associated with IκB to inhibit nuclear factor-κB after mechanical stimulation (Tomita et al., 2005). The importance of PPARγ in muscle differentiation was also reported with the connection to MyoD (Solanes et al., 2003). These results suggested that the intracellular levels of PPARγ were important in the progression of myogenesis. C2C12 cells treated with PPARγ agonist showed inhibited myogenesis, whereas a decrease in PPARγ levels promoted cell differentiation.

The stretch could enhance myosin expression and restore the retarded myogenesis caused by PPARγ activation. The PPRE promoter assay can detect the transcriptional activity of all different isoforms of PPARs (Liou et al., 2008). In the current study, there were no significant differences in the expression levels of PPARα and PPARδ. Therefore, the reduction of transcriptional activity may result from the decrease of PPARγ protein expression and cause a feedback loop to negatively regulate its signaling. The increase of PPRE activity induced by adding TZD into the MM was abolished by cyclic stretch (Figure 5B). These findings suggested that the mechanical stretch may cause the dissociation of PPRE binding. Much evidence has shown that exercise, a type of mechanical stimulation, inhibits adipose tissue formation and increases muscle tissue mass (Tanabe et al., 2004).

## Conclusion

This study explored the effects of mechanical forces on the cellular effects of PPARγ-related signaling and may explain the mechanical regulation of muscle mass on a microscopic scale. Therefore, the mechanical benefits of regulating PPARγ using cyclic stretch or exercise can be properly addressed in muscle homeostasis.

## Author Contributions

Authors read and approved this final manuscript. Y-J Chang collected the data, performed statistical analysis, and participated in manuscript preparation. Y-J Chen and C-WH helped to conduct the molecular biology experiments. S-CF assisted in the study design and thesis writing. W-TC, Y-ST, and F-CS provided technical support for experiments. C-CW is the corresponding author and contributed to the experimental design, article writing, and resource coordination.

## Conflict of Interest Statement

The authors declare that the research was conducted in the absence of any commercial or financial relationships that could be construed as a potential conflict of interest. The reviewer, C-LC, declares that, despite being affiliated to the same institution as the author, S-CF, the review process was handled objectively and no conflict of interest exists.

## Abbreviations

α-SMA, α-smooth muscle actin; DM, diabetes mellitus; DMEM, Dulbecco’s modified Eagle’s medium; ERK, extracellular signal-regulated kinase; GM, growth medium; JNK, c-Jun N-terminal kinase; MAPK, mitogen-activated protein kinase; MHC, myosin heavy chain; MM, myogenesis medium; PPAR, peroxisome proliferator-activated receptor; PPRE, PPAR response element; RSG, rosiglitazone; SF, serum-free DMEM; TZD, thiazolidinedione.
