# Supplementary figures and images for: Cyclic Stretch Facilitates Myogenesis in C2C12 Myoblasts and Rescues Thiazolidinedione-Inhibited Myotube Formation
Source: Front Bioeng Biotechnol. 2016 Mar 21;4:27. doi: 10.3389/fbioe.2016.00027 (PMC4800178; doi:10.3389/fbioe.2016.00027)

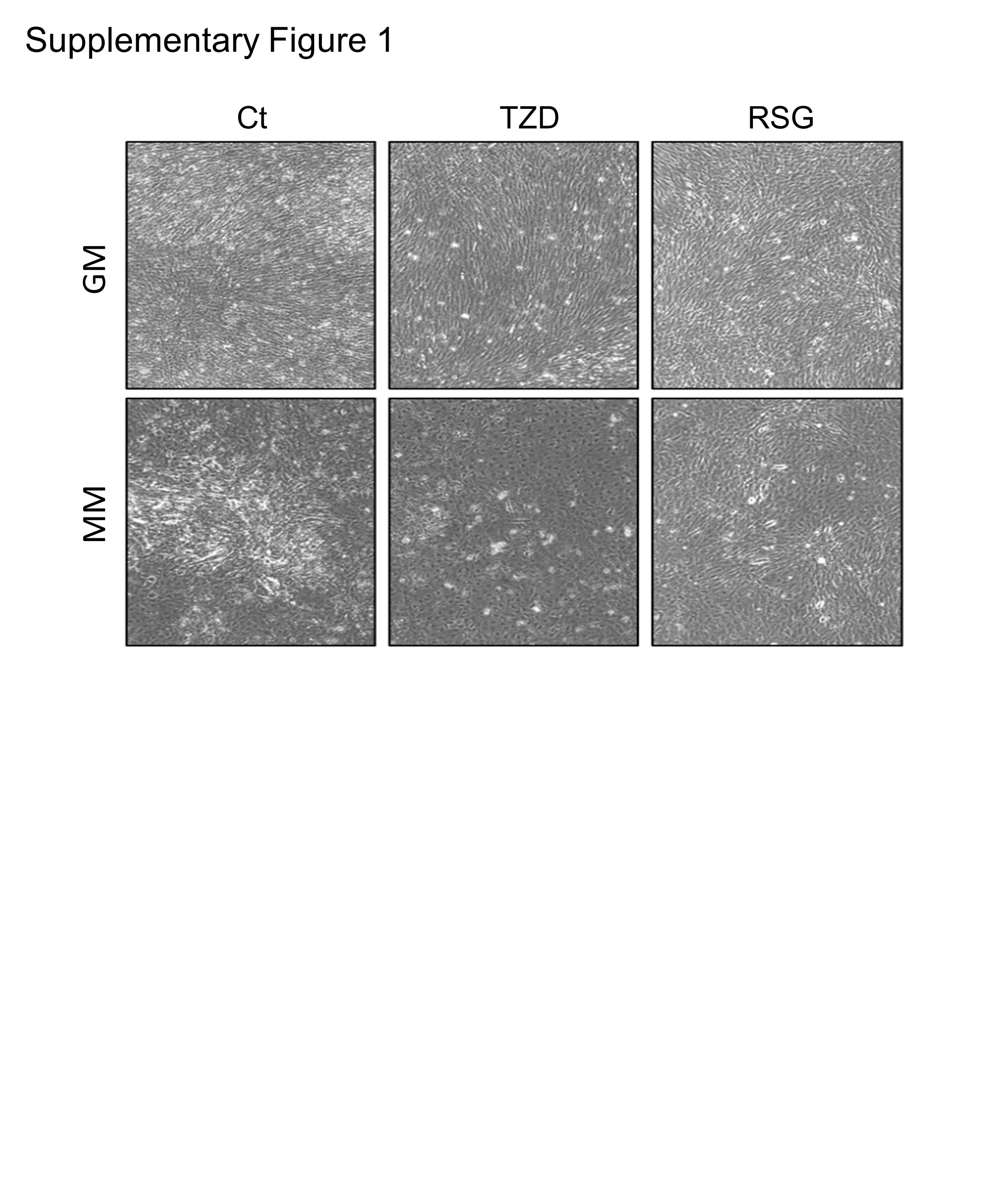

Supplement: Supplementary file 1 [file Image_1.JPEG]

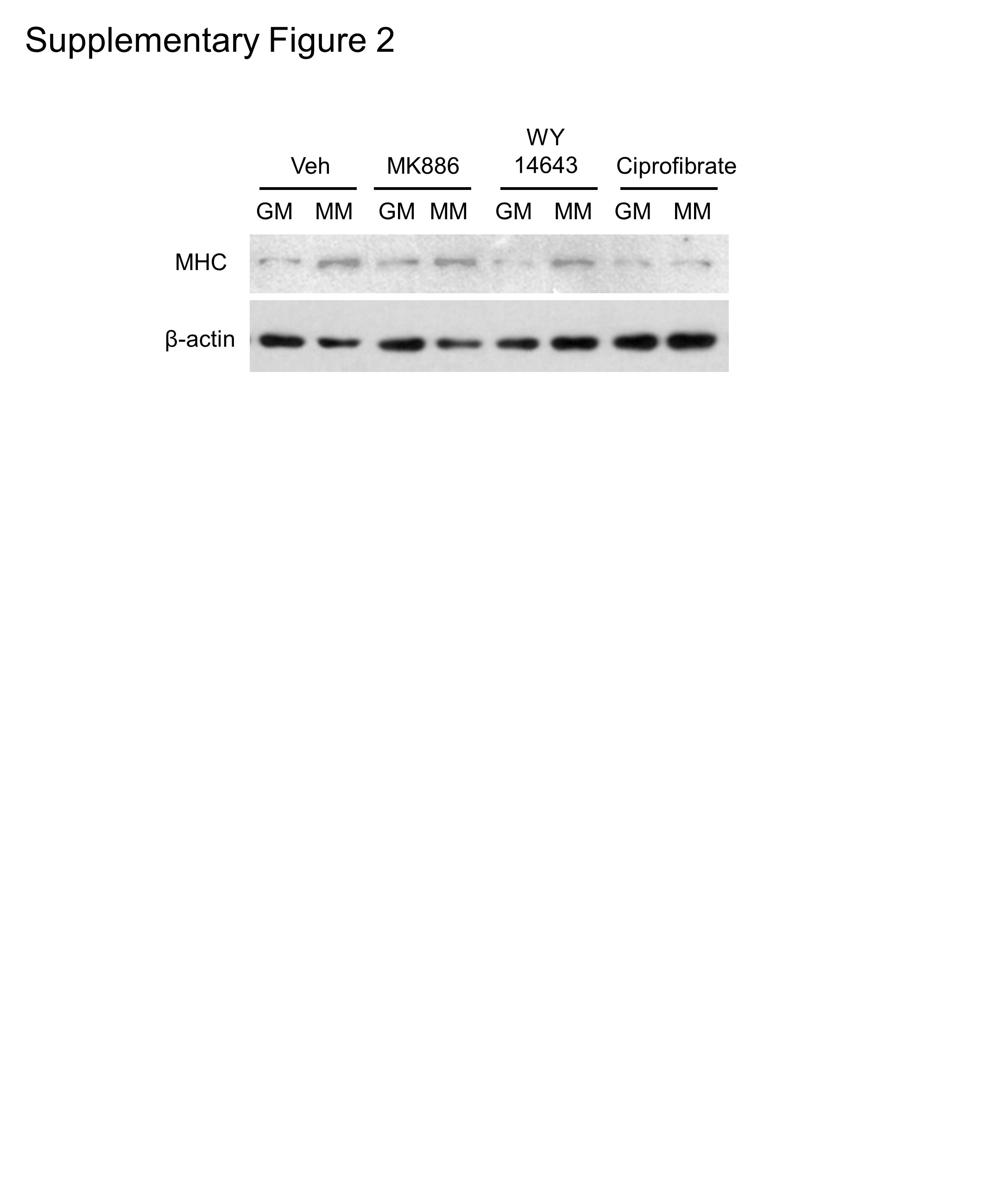

Supplement: Supplementary file 2 [file Image_2.JPEG]
